# Differences in prevalence of hypertension subtypes according to the 2018 Korean Society of Hypertension and 2017 American College of Cardiology/American Heart Association guidelines: The Korean National Health and Nutrition Examination Survey, 2007–2017 (KNHANES IV-VII)

**DOI:** 10.1186/s40885-019-0129-5

**Published:** 2019-12-01

**Authors:** So Mi Jemma Cho, Hokyou Lee, Hyeon Chang Kim

**Affiliations:** 1 Department of Public Health, Yonsei University Graduate School, Seoul, Korea; 2 Department of Preventive Medicine, Yonsei University College of Medicine, Seoul, 03722 Republic of Korea; 3 Department of Internal Medicine, Yonsei University College of Medicine, Seoul, Korea

**Keywords:** Hypertension, Blood pressure, Prevalence, Guideline, Cardiovascular diseases

## Abstract

**Background:**

The significance of high systolic and diastolic blood pressure remains controversial. We assessed the differences in prevalence of hypertension and its subtypes according to the different hypertension diagnostic criteria embodied by the 2017 American College of Cardiology/American Heart Association (2017 ACC/AHA) and 2018 Korean Society of Hypertension (2018 KSH) guidelines.

**Methods:**

We used the 2007–2017 Korea National Health and Nutrition Examination Survey (KNHANES) data to calculate guideline-specific hypertension prevalence among untreated, adult participants. By the 2017 ACC/AHA guideline, a mean SBP ≥130 mmHg, DBP ≥80 mmHg, or currently using antihypertensive medications were considered to have hypertension. Isolated diastolic hypertension (IDH) was defined as DBP ≥80 mmHg and SBP <130 mmHg, isolated systolic hypertension (ISH) as SBP ≥130 mmHg and DBP <80 mmHg, and systolic diastolic hypertension (SDH) as SBP ≥130 mmHg and DBP ≥80 mmHg. In a similar manner, by the 2018 KSH guideline, all hypertension and its subtype prevalence were calculated using the 140/90 mmHg cutoff. The two versions of all hypertension and its corresponding subtype prevalence were calculated among all study participants and separately by sex and age then compared via analysis of variance.

**Results:**

The prevalence of all hypertension increased from 25.9% (95% confidence interval (CI) 25.4–26.5) defined by the 2018 KSH guideline to 46.3% (95% CI 45.6–46.9) classified by the 2017 ACC/AHA guideline. Such increase was primarily manifested through substantial increase in IDH prevalence, from 5.2% (95% CI 4.9–5.4) defined by the 2018 KSH guideline to 17.9% (95% CI 17.4–18.3) defined by the 2017 ACC/AHA guideline, and was most notably observed in young age groups, 30-49 years. ISH prevalence showed minimal differences. SDH prevalence moderately increased from 3.5% (95% CI 3.3–3.7) defined by the 2018 KSH guideline to 11.1% (95% CI 10.7–11.4) defined by the 2017 ACC/AHA guideline, achieved primarily among participants aged 50 years or above.

**Conclusions:**

Changes in each subtype prevalence made differential contribution to additionally classified hypertension cases by the 2017 ACC/AHA guideline. Future studies should investigate the diastolic-associated cardiovascular risks and benefits of its long-term primary prevention in the young population.

## Introduction

High blood pressure (BP) is a major yet modifiable risk factor for cardiovascular diseases (CVD) [1–4]. Despite its established causal and exacerbating role in a wide array of chronic diseases, high BP remains a global concern, contributing to substantial morbidity and mortality [5–8]. Clinical and epidemiological studies have demonstrated continuous association between high BP and CVD risk in all sex, race, and age [9–11]. However, considering the different types and distributions of CVD risk factors across different demographics [12], previous studies have reported diverging findings regarding the significance of elevated systolic and diastolic blood pressure (SBP & DBP) [13–15]. One meta-analysis showed that each 20-mmHg SBP or 10-mmHg DBP increase was associated with approximately a two-fold increased risk for stroke and vascular-related mortality without definite lower threshold [9]. Yet, a recent analysis of the Korean national health insurance data indicated while elevated SBP remains an independent and strong predictor of future CVD, DBP showed SBP-dependent indication [16]. Accounting for diverging claims on role of elevated SBP versus DBP, it deems essential to evaluate the nation’s recent prevalence of hypertension, specifically by its subtypes.

With continued effort to provide optimal prevention and management guideline on hypertension, the 2017 American College of Cardiology/ American Heart Association Guideline for the Prevention, Detection, Evaluation, and Management of High Blood Pressure in Adults (2017 ACC/AHA guideline) [17] have adopted a markedly lower BP criteria in defining hypertension, from the Seventh Report of the Joint National Committee on Prevention, Detection, Evaluation and Treatment of High Blood Pressure (JNC 7) [18]’s previous 140/90 mmHg to 130/80 mmHg. Despite the diagnostic criteria remained 140/90 mmHg, the 2018 Korean Society of Hypertension Guidelines for the Management of Hypertension (2018 KSH guideline) [19] considered recommendation for pharmacological treatment in prehypertension (130–139 mmHg/80–89 mmHg) individuals with high-risk for atherosclerotic cardiovascular diseases (ASCVD), such as chronic kidney disease (CKD) or diabetes mellitus (DM) comorbidity.

Timely assessment of pervasiveness of hypertension and its management across the entire lifespan are crucial in preventing further adverse health outcomes at individual level and conserving substantial healthcare resources at national level. With implementation of the new diagnostic criteria in 2017 ACC/AHA guideline and increasing emphasis on future ASCVD risk level observed in the most recent Korean guideline, our objective was to assess the difference in prevalence of hypertension and its subtypes by sex and age groups according to the two aforementioned guidelines. Specifically, by investigating which subtype and population characteristics are primarily responsible for overall hypertension prevalence difference, we aimed to provide framework in detecting potential target group that will benefit from early lifestyle and/or pharmaceutical interventions in contemporary Korean population.

## Methods

### Study population

This study analyzed data from the Korea National Health and Nutrition Examination Survey (KNHANES) conducted between 2007 and 2017. The KNHANES is an ongoing surveillance system in the Republic of Korea that assesses current health status and distributions of chronic diseases risk factors [20]. It collects detailed information on demographics, disease history, health behaviors, and healthcare utilization, nutrition and provides detailed anthropometric, blood, and urinal profiles from on-site health examination [20]. To produce unbiased cross-sectional estimates of the Korean population, it uses complex, multi-stage probability sample design. Sample weights are constructed to account for non-response and post-stratification [20]. The details of the KNHANES is published elsewhere [20, 21]. The KNHANES IV-VII were administered by the Korea Center for Disease Control and Prevention and approved by the Institutional Review Board (2007–02-CON-04-P, 2008-04EXP-01-C, 2009-01CON-03-2C, 2010-02CON-21-C, 2011-02CON-06-C, 2012-01EXP-01-2C, 2013-07CON-03-4C, 2013-12EXP-03-5C, 2015-01-02-6C). In the present study, among 89,630 participants, 21,473 child/adolescent participants under the age of 20 were excluded. Then, 4853 participants with missing BP measurements or information on antihypertensive medication intake were further excluded. Of the remaining 63,304 participants, 3537 participants without measurement on key covariates required to calculate ASCVD risk score, including cigarette smoking history, DM prevalence, and total and high-density lipoprotein cholesterols were additionally excluded. Consequently, 59,767 participants (26,920 male and 36,384 female) were included for the final analyses.

### Blood pressure measurement and definition of hypertension and its subtypes

BP was measured using a standard mercury sphygmomanometer (Baumanometer Wall Unit 33(0850); Baum Co., Inc., Copiague, NY, USA). Participants sat in a comfortable position after they had rested for at least 5 min and had refrained from smoking 30 min prior to the measurement. BP was measured on three consecutive occasions in a standardized environment, free of disturbances, at 1-min intervals. The average of the second and the third measurements was adopted for the data analysis. Information regarding hypertension treatment was obtained via self-report. Participants who used antihypertensive medication for 20 or more days per month were classified as the treated group. Hypertension prevalence was defined according to the 2017 ACC/AHA as well as the 2018 KSH guidelines among total study participants.

According to the 2017 ACC/AHA guideline, participants with SBP ≥130 mmHg, DBP ≥80 mmHg, or current antihypertensive medication were considered to have hypertension. Then, among participants with untreated hypertension, three types of hypertension subtypes were operationally defined and assessed: isolated diastolic hypertension (IDH) was defined as SBP <130 mmHg and DBP ≥80 mmHg; isolated systolic hypertension (ISH) was defined as SBP ≥130 mmHg and DBP <80 mmHg; systolic diastolic hypertension (SDH) was defined as SBP ≥130 mmHg and DBP ≥80 mmHg. Likewise, according to the 2018 KSH guideline, hypertension was defined as SBP ≥140 mmHg, DBP ≥90 mmHg, or current intake of antihypertensive medications. Again, we further stratified the untreated individuals into one of the following subtypes: IDH, defined as SBP <140 mmHg and DBP ≥90 mmHg, ISH defined as SBP ≥140 mmHg and DBP <90 mmHg, and SDH as SBP ≥140 mmHg and DBP ≥90 mmHg.

The guideline-specific prevalence of all hypertension and its corresponding subtypes were calculated among all study participants combined and separately by sex and decile age groups. All presented statistics are weighted accordingly to account for contemporary Korean population structure.

### Covariates

By cross-referencing with government issued identification code, we ensured participants’ biological age in years, then categorized into: 20–29 years, 30–39 years, 40–49 years, 50–59 years, 60–69 years, 70–79 years, and 80 years or above. Using validated questionnaire, trained interviewee collected information on the following. Physical activity was assessed by fulfillment of “sufficient activity” by the Korean version of the International Physical Activity Questionnaire standard [22]. Alcohol consumption and cigarette smoking status was divided into current-, previous-, and non- by the time of survey administration. History of chronic diseases, including formal diagnosis by medical doctor and treatment status was also collected.

The health examination obtained participants’ body weight and height to the nearest 0.1 kg and 0.1 cm, respectively, while wearing light clothing without shoes. Body mass index was then calculated as the ratio of weight in kilograms to height in squared meters. For chemistry tests, cholesterol and fasting glucose concentrations were enzymatically assessed via Hitachi Automatic Analyzer 7600 (Hitachi, Tokyo, Japan) in 2007 and COBAS 8000 C702 (Roche Diagnostics System, Rotkreuz, Switzerland) onwards. In accordance with the 2019 Clinical Practice Guidelines for Type 2 Diabetes Mellitus in Korea [23] 8-h fasting plasma blood glucose ≥126 mg/dL, glycated hemoglobin level ≥6.5%, or current DM treatment was considered prevalent DM. From 8-h fasting cholesterols and 12-h fasting triglyceride, we referred to the Korean Society of Lipid and Atherosclerosis’ 2018 Guidelines for The Management of Dyslipidemia [24]: hypercholesterolemia referred to total cholesterol ≥240 mg/dL; hypertriglyceridemia referred to triglyceride ≥200 mg/dL; hypo-high-density-lipoprotein-cholesterolemia referred to high-density lipoprotein cholesterol <40 mg/dL; or current lipid-lowering treatment. Using serum creatinine concentration, glomerular filtration rate was derived from the Chronic Kidney Disease-Epidemiology Collaboration [25] equation. Based on Kidney Disease Improving Global Outcomes 2018 guideline [26], glomerular filtration rate <60 mL/min/1.73 m^2^ was considered prevalent CKD. To assess whether differences in hypertension prevalence are accompanied by differences in predicted cardiovascular risk, we yielded 10-year ASCVD risk scores that were derived from two different algorithms: 1) 2013 ACC/AHA Pooled Cohort Equation (PCE) [27]; 2) Korean Risk Prediction Model (KRPM) [28]. Quality of the survey was controlled by trained personnel using calibrated equipment and strictly adhering to standardized protocols.

### Statistical analyses

General characteristics of the study population were reported as weighted frequency or mean and its corresponding 95% confidence interval (CI). Differences in participant characteristics across stages of hypertension and by the hypertension subtypes were compared via analysis of variance. All hypertension prevalence calculated by dividing the number of pertinent (subtype) cases by the total study population. The same was done after stratifying the participants by sex and age groups. All statistical tests were two-sided, and statistical significance was set at a *p*-value <0.05. All analyses were performed using SAS version 9.4 (SAS Institute Inc., Cary, NC).

## Results

### Participant characteristics

Table 1 presents general characteristics of participants by BP classification according to the 2017 ACC/AHA and 2018 KSH guidelines, separately. The treated individuals are allocated in a separate group. Regardless of the referred guideline, participants classified into higher stage hypertension were older with higher proportion of male sex. In general, they also had higher body mass index, glycemic and lipid indices and more reduced kidney function. When examining health-related lifestyle, participants with higher BP were more likely to be current smoker and drinker yet less likely to fulfill regular physical activity. Altogether, participants with higher BP embodied higher predicted 10-year ASCVD risk score by both the PCE (2.0% in normal BP vs. 19.1% in stage 2 hypertension by 2018 KSH guideline and 16.4% by 2017 ACC/AHA guideline) and KRPM (2.0% in normal BP vs. 15.5% in stage 2 hypertension by 2018 KSH guideline and 12.2% by 2017 ACC/AHA guideline), respectively.

**Table 1:** Characteristics of study participants by 2017 ACC/AHA and 2018 KSH guidelines and antihypertensive medication use

	Normal BP;	Elevated BP;	2018 KSH Guideline			2017 ACC/AHA Guideline		Treated HTN
			Prehypertension;	Stage 1 HTN;	Stage 2 HTN;	Stage 1 HTN;	Stage 2 HTN;	
	SBP <120 mmHg	SBP 120–129 mmHg	SBP 130–139 mmHg	SBP 140–159 mmHg	SBP ≥160 mmHg	SBP 130–139 mmHg	SBP ≥140 mmHg	
	&	&	or	or	or	or	or	
	DBP <80 mmHg	DBP < 80 mmHg	DBP 80–89 mmHg	DBP 90–99 mmHg	DBP ≥100 mmHg	DBP 80–89 mmHg	DBP ≥90 mmHg	
	(*n* = 26,648)	(*n* = 3406)	(*n* = 11,336)	(*n* = 5288)	(*n* = 1392)	(*n* = 11,336)	(*n* = 6680)	(*n* = 11,697)
% Study population	48.5 (47.9–49.2)	5.3 (5.0–5.5)	20.4 (19.9–20.8)	8.8 (8.5–9.1)	2.3 (2.2–2.5)	20.4 (19.9–20.8)	11.1 (10.8–11.5)	14.7 (14.3–15.1)
Male sex	40.1 (39.5–40.8)	54.7 (52.8–56.7)	64.2 (63.2–65.2)	66.9 (65.5–68.3)	70.5 (67.8–73.2)	64.2 (63.2–65.2)	67.7 (66.4–68.9)	47.6 (46.5–48.7)
Age, yrs	39.7 (39.5–39.9)	49.4 (48.6–50.3)	45.0 (44.6–45.3)	49.6 (49.1–50.1)	50.0 (49.2–50.8)	45.0 (44.6–45.3)	49.7 (49.3–50.1)	62.7 (62.5–63.0)
Smoking status								
Non-smoker	61.0 (30.2–61.6)	54.5 (52.3–56.6)	45.4 (44.3–46.5)	42.5 (40.9–44.1)	37.0 (34.1–39.9)	45.4 (44.3–46.5)	41.3 (39.9–42.7)	55.9 (54.8–57.0)
Previous smoker	12.1 (11.6–12.6)	16.3 (14.7–17.9)	17.9 (17.0–18.8)	18.1 (16.8–19.4)	22.0 (19.2–24.9)	17.9 (17.0–18.8)	18.9 (17.7–20.1)	21.6 (20.6–22.5)
Current smoker	27.0 (26.3–27.7)	29.2 (27.2–31.3)	36.7 (35.6–37.9)	39.4 (37.8–41.1)	41.0 (38.0–43.9)	36.7 (35.6–37.9)	39.8 (38.3–41.2)	22.5 (21.5–23.5)
Alcohol intake								
Non-drinker	7.7 (7.3–8.1)	12.4 (11.2–16.7)	8.2 (7.7–8.8)	9.8 (9.0–10.7)	8.7 (7.1–10.2)	8.2 (7.7–8.8)	9.6 (8.8–10.3)	19.6 (18.8–20.5)
Former drinker	34.0 (33.3–34.7)	32.2 (30.2–34.1)	26.3 (25.3–27.3)	22.5 (21.2–23.9)	19.6 (17.3–22.0)	26.3 (25.3–27.3)	21.9 (20.7–23.1)	32.5 (31.5–33.5)
Current drinker	58.3 (57.5–59.0)	55.4 (53.3–57.5)	65.5 (64.4–66.6)	67.7 (66.2–69.2)	71.7 (69.0–74.4)	65.5 (64.4–66.6)	68.5 (67.2–69.9)	47.9 (46.7–49.0)
Regular exercise								
Yes	28.6 (28.0–29.5)	29.9 (27.7–32.2)	30.0 (28.8–31.2)	28.8 (27.1–30.5)	26.9 (23.9–29.8)	30.0 (28.8–31.2)	28.4 (26.9–29.9)	24.4 (23.2–25.5)
No	71.4 (70.5–72.2)	70.1 (67.8–72.3)	70.0 (68.8–71.2)	71.2 (69.5–72.9)	73.1 (70.2–76.1)	70.0 (68.8–71.2)	71.6 (70.1–73.1)	75.6 (74.5–76.8)
BMI, kg/m^2^	22.7 (22.7–22.8)	24.0 (23.9–24.2)	24.4 (24.3–24.5)	25.1 (24.9–25.2)	25.5 (25.3–25.8)	24.4 (24.3–24.5)	25.2 (25.0–25.3)	25.3 (25.2–25.4)
Total cholesterol, mg/dL	183.8 (183.3–184.3)	190.6 (189.1–192.1)	196.0 (195.2–196.8)	200.0 (198.7–201.3)	203.6 (201.4–205.8)	196.0 (195.2–196.8)	200.8 (199.6–201.9)	187.0 (186.1–187.8)
HDL cholesterol, mg/dL	51.7 (51.6–51.9)	49.4 (48.9–49.9)	49.1 (48.9–49.4)	48.5 (48.1–48.9)	48.2 (47.5–48.9)	49.1 (48.9–49.4)	48.4 (48.1–48.8)	46.9 (46.6–47.1)
Triglyceride, mg/dL	110.9 (109.7–112.2)	137.8 (133.1–142.5)	158.0 (154.5–161.5)	178.0 (172.3–183.6)	194.4 (184.6–204.3)	158.0 (154.5–161.5)	181.4 (176.5–186.4)	160.2 (157.5–163.0)
Lipid-lowering agent intake	1.9 (1.8–2.1)	5.5 (4.7–6.4)	2.8 (2.5–3.2)	2.9 (2.4–3.4)	1.7 (0.9–2.4)	2.8 (2.5–3.2)	2.6 (2.2–3.1)	24.4 (23.4–25.3)
Fasting glucose, mg/dL	93.1 (92.8–93.3)	100.4 (99.4–101.3)	99.0 (98.5–99.6)	102.0 (101.2–102.8)	104.9 (103.0–106.9)	99.0 (98.5–99.6)	102.6 (101.9–103.3)	109.4 (108.8–110.1)
GFR, ml/min/1.73 m^2^	101.8 (101.4–102.1)	94.6 (93.9–95.4)	96.3 (95.9–96.7)	93.2 (92.6–93.7)	91.8 (90.8–92.8)	96.3 (95.9–96.7)	92.9 (92.4–93.4)	81.9 (81.5–82.3)
Diabetes mellitus	4.0 (3.7–4.2)	11.5 (10.3–12.8)	7.8 (7.2–8.3)	10.1 (9.1–11.0)	10.7 (8.7–12.7)	7.8 (7.2–8.3)	10.2 (9.3–11.0)	71.8 (70.8–72.9)
Chronic Kidney Disease	0.7 (0.6–0.8)	2.5 (1.9–3.0)	1.0 (0.8–1.2)	2.4 (2.0–2.9)	2.9 (1.9–3.8)	1.0 (0.8–1.2)	2.5 (2.1–2.9)	11.1 (10.4–11.8)
Mean 10-yr predicted ASCVD risk^*^								
High risk,^*^%, PCE	2.0 (1.8–2.2)	16.0 (14.6–17.3)	6.5 (6.0–7.0)	14.6 (13.6–15.6)	19.1 (16.9–21.3)	6.5 (6.0–7.0)	15.5 (14.6–16.5)	88.9 (88.2–89.6)
High risk, ^*^%, KRPM	2.3 (2.2–2.5)	11.8 (10.7–12.9)	5.5 (5.1–5.9)	11.3 (10.4–12.2)	17.2 (15.0–19.4)	5.5 (5.1–5.9)	12.5 (11.6–13.4)	88.9 (88.2–89.6)
History of CVD	2.4 (2.2–2.6)	6.9 (5.9–8.0)	4.3 (3.8–4.8)	4.7 (3.9–5.4)	2.7 (1.9–3.5)	4.3 (3.8–4.8)	4.3 (3.7–4.9)	21.3 (20.2–22.4)

Values are presented as weighted % or mean (95% confidence interval)

The study participants were grouped into the higher category of SBP and DBP. For example, if a person had SBP of 146 mmHg and DBP of 82 mmHg, they were grouped into the ≥140/90 mmHg category

*Abbreviations*: *ACC/AHA* American College of Cardiology/American Heart Association, *BMI* Body mass index, *CI* Confidence interval, *CKD* Chronic kidney disease, *CVD* Cardiovascular disease, *GFR* Glomerular filtration rate, *HDL* High-density lipoprotein, *HTN* Hypertension, *KRPM* Korean Risk Prediction Model, *KSH* Korean Society of Hypertension, *PCE* Pooled Cohort Equation

^*^10-yr risk was calculated among adults without a history of CVD

^*^High risk defined as a 10-year predicted cardiovascular disease risk ≥10% or history of CVD

### Prevalence of hypertension and its subtypes

**Fig. 1:**
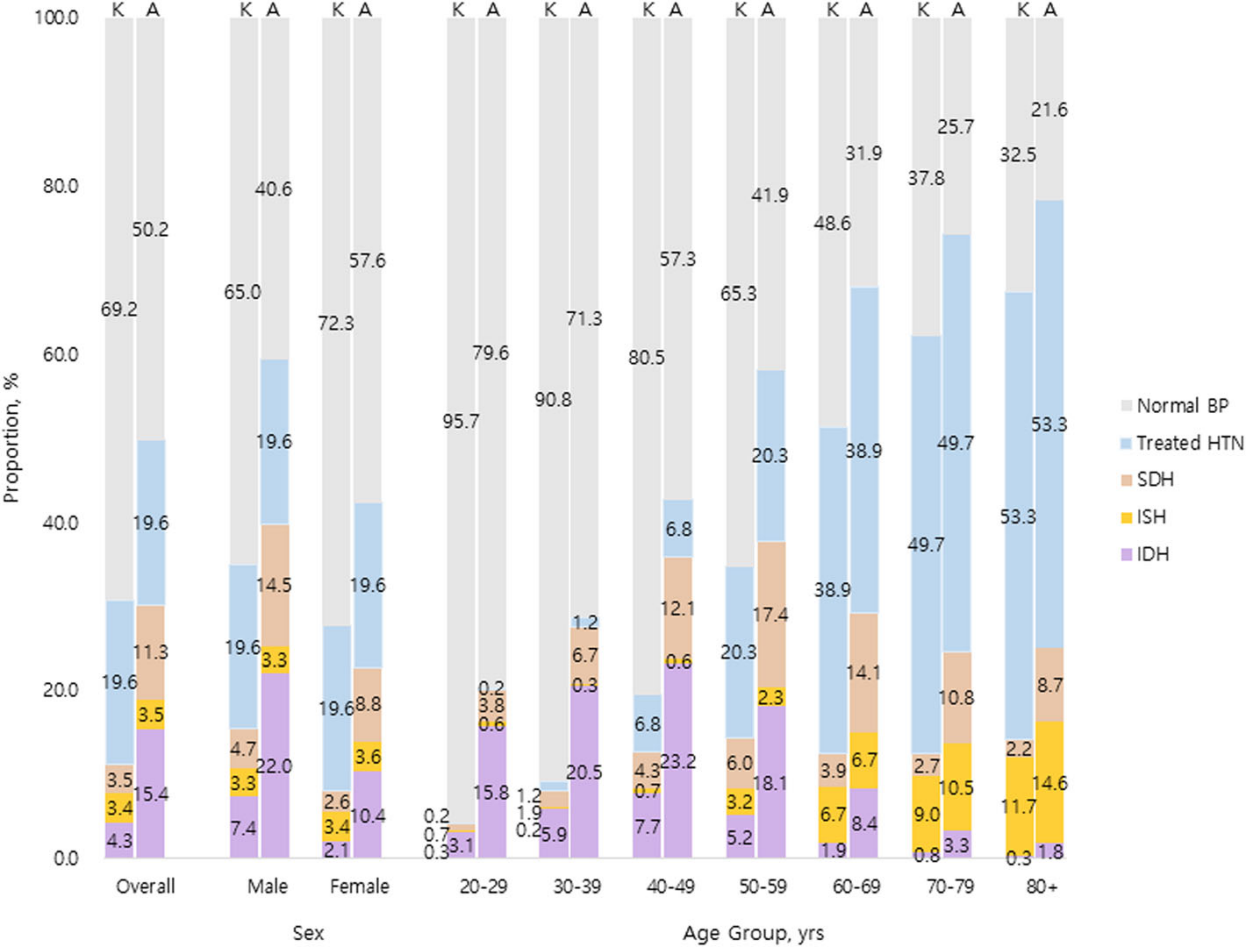
Hypertension subtype prevalence according to the 2017 ACC/AHA and 2018 KSH guidelines in total population. Abbreviations: ACC/AHA, American College of Cardiology/American Heart Association; BP, blood pressure; HTN, hypertension; IDH, isolated diastolic hypertension; ISH, isolated systolic hypertension; KSH, Korean Society of Hypertension; SDH, systolic diastolic hypertension

**Fig. 2:**
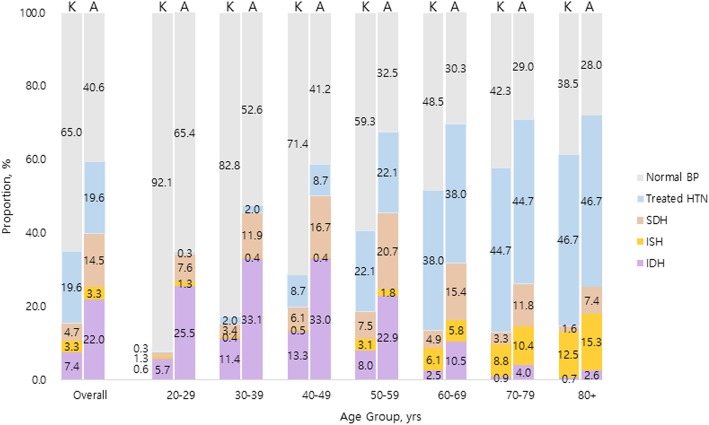
Hypertension subtype prevalence according to the 2017 ACC/AHA and 2018 KSH guidelines in male population. Abbreviations: ACC/AHA, American College of Cardiology/American Heart Association; BP, blood pressure; HTN, hypertension; IDH, isolated diastolic hypertension; ISH, isolated systolic hypertension; KSH, Korean Society of Hypertension; SDH, systolic diastolic hypertension

**Fig. 3:**
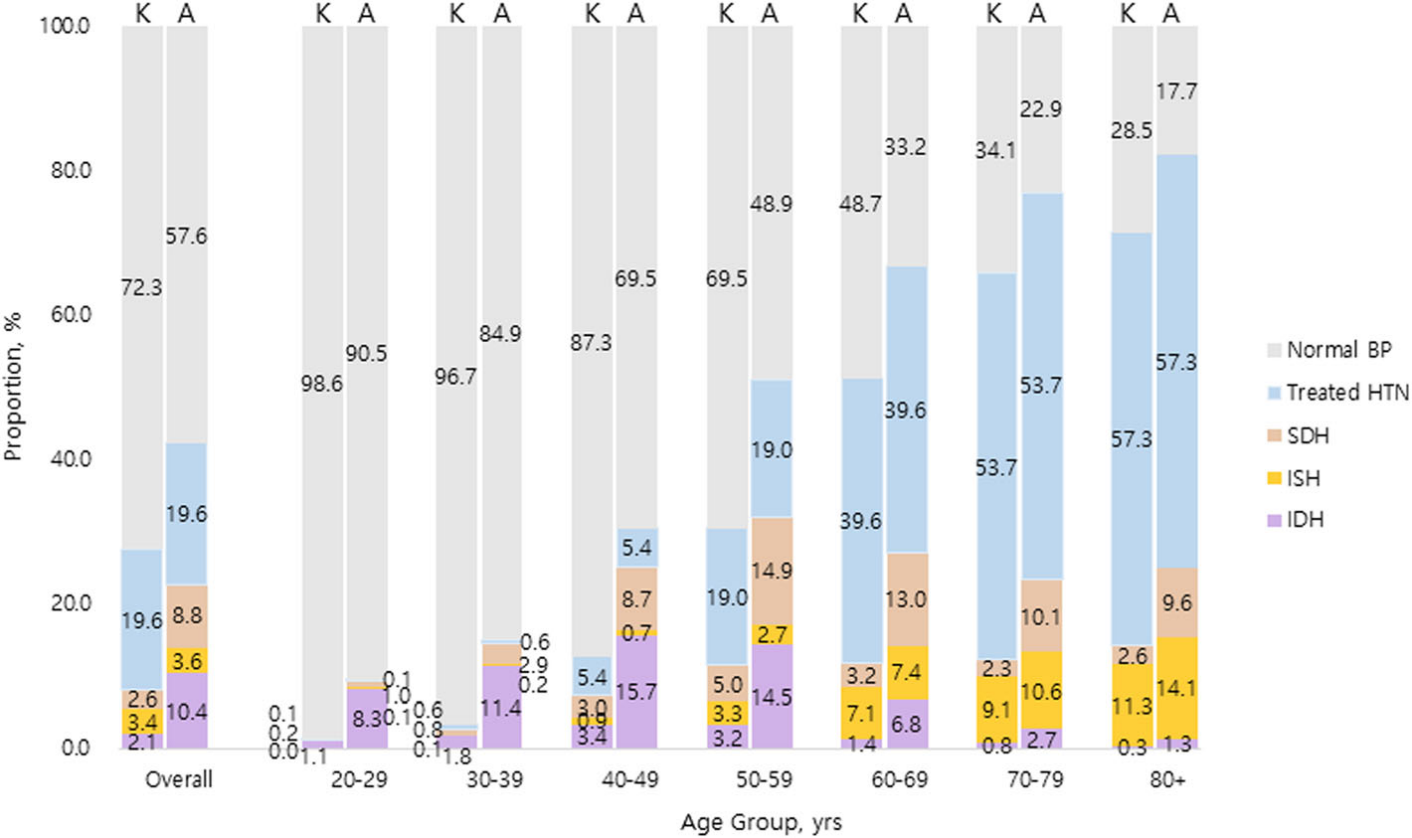
Hypertension subtype prevalence according to the 2017 ACC/AHA and 2018 KSH guidelines in female population. Abbreviations: ACC/AHA, American College of Cardiology/American Heart Association; BP, blood pressure; HTN, hypertension; IDH, isolated diastolic hypertension; ISH, isolated systolic hypertension; KSH, Korean Society of Hypertension; SDH, systolic diastolic hypertension

Sex- and age-stratified all hypertension and subtype prevalence are illustrated in Table 2 and Figs. 1, 2 and 3. In general, the degree of difference varied in each subtype by the guideline used for BP classification. Overall, the prevalence of all hypertension increased from 25.9% (95% CI 25.4–26.5) defined by the 2018 KSH guideline to 46.3% (95% CI 45.6–46.9) classified by the 2017 ACC/AHA guideline, the latter yielding additional 20.3%. The addendum was most distinguished in age group 40–49 years (23.9%, 95% CI 23.0–24.8); in sex-stratified analysis, such trend was retained in male (30.1%, 95% CI 28.6–31.5) whereas observed in older female, 50–59 years (20.5%, 95% CI 19.3–21.7). Yet, in both sex, the additional inclusion of newly diagnosed hypertension by the 2017 ACC/AHA guideline primarily came from younger participants. Else, 14.7% (95% CI 14.3–15.1) of the participants were currently receiving pharmaceutical treatment for hypertension regardless of the guideline referred to.

**Table 2:** Prevalence of hypertension and its subtypes according to the 2017 ACC/AHA and 2018 KSH guidelines

	All HTN			Treated HTN	Untreated isolated diastolic HTN			Untreated isolated systolic HTN			Untreated systolic diastolic HTN		
	2018 KSH	2017 ACC/AHA	Difference	All guideline	2018 KSH	2017 ACC/AHA	Difference	2018 KSH	2017 ACC/AHA	Difference	2018 KSH	2017 ACC/AHA	Difference
	(*n* = 18,377)	(*n* = 29,713)	(*n* = 11,336)	(*n* = 11,697)	(*n* = 2592)	(*n* = 9191)	(*n* = 6593)	(*n* = 2007)	(*n* = 2082)	(*n* = 79)	(*n* = 2081)	(*n* = 6743)	(*n* = 4627)
Total	25.9 (25.4–26.5)	46.3 (45.6–46.9)	20.3 (19.9–20.8)	14.7 (14.3–15.1)	5.2 (4.9–5.4)	17.9 (17.4–18.3)	11.8 (11.5–12.1)	2.5 (2.3–2.6)	2.6 (2.4–2.7)	0.1 (0.0–0.2)	3.5 (3.3–3.7)	11.1 (10.7–11.4)	7.6 (7.3–7.8)
Age group, yrs													
20–29	4.8 (4.2–5.4)	22.5 (21.3–23.8)	17.7 (16.6–18.9)	0.2 (0.1–0.4)	3.5 (3.0–4.1)	17.2 (16.1–18.3)	12.5 (12.3–12.7)	0.4 (0.2–0.5)	0.7 (0.5–0.9)	0.3 (0.2–0.4)	0.7 (0.5–1.0)	4.4 (3.8–5.0)	3.7 (3.2–4.3)
30–39	10.3 (9.6–11.0)	31.6 (30.5–32.7)	21.3 (20.4–22.3)	1.8 (1.5–2.2)	6.5 (6.0–7.1)	22.4 (21.4–23.4)	14.9 (14.7–15.2)	0.3 (0.1–0.4)	0.4 (0.2–0.5)	0.1 (0.0–0.2)	2.2 (1.8–2.5)	7.5 (6.9–8.1)	5.3 (4.8–5.9)
40–49	21.1 (20.2–22.0)	45.0 (43.9–46.1)	23.9 (23.0–24.8)	10.8 (9.9–11.6)	8.5 (7.9–9.1)	24.3 (23.3–25.3)	15.2 (15.0–15.4)	0.7 (0.5–0.9)	0.6 (0.4–0.8)	−0.1 (− 0.2–0.0)	4.6 (4.2–5.1)	12.9 (12.2–13.7)	8.3 (7.7–8.9)
50–59	35.3 (34.3–36.4)	59.1 (58.1–60.2)	23.8 (22.9–24.7)	26.1 (25.0–27.1)	5.5 (5.0–6.0)	18.9 (18.0–19.7)	13.1 (12.8–13.3)	3.2 (2.8–3.6)	2.3 (1.9–2.6)	− 0.9 (− 1.0--0.8)	6.4 (5.8–6.9)	18.9 (18.0–19.7)	11.4 (10.7–12.1)
60–69	51.1 (49.9–52.3)	67.8 (66.7–69.0)	16.7 (15.8–17.6)	30.0 (29.0–31.0)	1.9 (1.6–2.2)	8.5 (7.8–9.1)	6.5 (6.1–6.8)	6.3 (5.7–7.0)	6.5 (5.9–7.1)	0.0 (0.0–0.0)	4.0 (3.5–4.4)	14.0 (13.2–14.9)	10.1 (9.3–10.8)
70–79	62.6 (61.2–63.9)	74.6 (73.4–75.8)	12.1 (11.1–13.0)	25.0 (24.1–25.9)	0.7 (0.5–0.9)	3.0 (2.6–3.5)	2.1 (1.8–2.4)	8.7 (8.0–9.5)	11.0 (10.2–11.9)	1.4 (1.3–1.6)	2.6 (2.2–3.1)	10.1 (9.3–11.0)	7.5 (6.8–8.2)
80+	69.7 (66.8–72.5)	79.8 (77.4–82.2)	10.1 (8.4–11.9)	6.1 (5.6–6.6)	0.5 (0.1–0.8)	1.6 (0.9–2.2)	1.6 (1.3–1.8)	12.1 (10.2–14.1)	15.0 (12.8–17.2)	2.8 (2.7–3.0)	2.2 (1.4–3.1)	8.8 (7.1–10.5)	6.6 (5.1–8.1)
Male	29.2 (2.4–29.9)	55.3 (54.4–56.1)	26.1 (25.4–26.8)	14.0 (13.5–14.5)	82 (7.8–8.7)	24.9 (24.1–25.6)	14.4 (14.0–14.7)	2.3 (2.1–2.5)	2.2 (2.0–2.4)	0.0 (0.0–0.0)	4.5 (4.2–4.9)	14.1 (13.6–14.7)	9.6 (9.1–10.0)
Age group, yrs													
20–29	7.9 (6.8–9.0)	34.1 (32.1–36.1)	26.2 (24.4–28.0)	0.5 (0.1–0.8)	5.7 (4.8–6.7)	25.0 (23.2–26.8)	19.6 (19.3–20.2)	0.7 (0.3–1.0)	1.2 (0.8–1.7)	0.6 (0.5–0.7)	1.2 (0.8–1.6)	7.5 (6.4–8.6)	6.3 (5.3–7.3)
30–39	16.8 (15.6–18.0)	46.8 (45.1–48.4)	30.0 (28.5–31.5)	2.9 (2.3–3.6)	11.0 (10.0–11.9)	32.5 (30.9–34.0)	21.5 (21.1–22.0)	0.4 (0.2–0.7)	0.5 (0.3–0.7)	0.0 (0.0–0.0)	3.4 (2.8–4.0)	11.8 (10.8–12.9)	8.4 (7.5–9.3)
40–49	29.0 (27.5–30.4)	59.0 (57.5–60.6)	30.1 (28.6–31.5)	13.9 (12.5–15.3)	13.3 (12.2–14.4)	33.0 (31.5–34.5)	19.5 (19.3–20.0)	0.6 (0.4–0.9)	0.4 (0.2–0.7)	− 0.1 (− 0.2–0.0)	6.3 (5.5–7.0)	16.9 (15.7–18.1)	10.6 (9.6–11.6)
50–59	40.4 (38.8–41.9)	67.4 (65.9–68.9)	27.0 (25.6–28.4)	29.3 (27.7–30.9)	7.9 (7.0–8.7)	23.1 (21.7–24.4)	15.5 (15.1–15.9)	3.3 (2.7–4.0)	1.9 (1.4–2.3)	− 1.4 (− 1.5--1.2)	7.6 (6.8–8.5)	20.9 (19.6–22.3)	13.3 (12.2–14.4)
60–69	51.2 (49.4–52.9)	69.2 (67.5–70.8)	18.0 (16.6–19.3)	29.9 (28.4–31.4)	2.5 (2.0–3.0)	10.3 (9.3–11.3)	7.9 (7.7–8.1)	5.5 (4.8–6.2)	5.4 (4.6–6.2)	− 0.3 (− 0.4−− 0.2)	4.8 (4.1–5.6)	15.1 (13.8–16.3)	10.3 (9.2–11.3)
70–79	58.1 (56.1–60.1)	71.1 (69.3–73.0)	13.0 (11.7–14.4)	19.6 (18.4–20.7)	0.7 (0.4–1.0)	4.0 (3.2–4.8)	3.4 (3.0–3.7)	8.9 (7.8–10.0)	11.0 (9.7–12.3)	1.8 (1.6–2.0)	3.1 (2.4–3.8)	10.8 (9.6–12.0)	7.7 (6.7–8.7)
80+	63.0 (58.3–67.8)	73.7 (69.4–78.0)	10.7 (7.8–13.6)	3.9 (3.4–4.5)	0.6 (0.0–1.3)	2.6 (1.1–4.1)	1.8 (1.6–2.0)	12.7 (9.6–15.8)	15.7 (12.4–19.0)	2.5 (2.3–2.8)	1.5 (0.5–2.5)	7.2 (4.7–9.7)	5.7 (3.4–8.0)
Female	22.7 (22.1–23.3)	37.3 (36.5–38.0)	14.5 (14.1–15.0)	15.4 (14.9–15.9)	2.1 (1.9–2.3)	10.9 (10.4–11.3)	8.5 (8.0–8.9)	2.6 (2.4–2.8)	2.9 (2.7–3.1)	0.2 (0.1–0.4)	2.4 (2.2–2.6)	8.0 (7.7–8.4)	5.6 (5.3–5.9)
Age group, yrs													
20–29	1.3 (0.9–1.7)	9.4 (8.3–10.5)	8.1 (7.1–9.1)	0.0 (0.0–0.1)	1.0 (0.7–1.4)	8.3 (7.3–9.3)	7.0 (6.7–7.3)	0.0 (0.0–0.0)	0.1 (0.0–0.2)	0.1 (0.0–0.2)	0.2 (0.0–0.4)	1.0 (0.6–1.3)	0.8 (0.5–1.1)
30–39	3.4 (2.9–3.9)	15.5 (14.4–16.6)	12.1 (11.2–13.1)	0.8 (0.6–1.1)	1.8 (1.5–2.2)	11.7 (10.8–12.7)	9.5 (9.3–9.8)	0.1 (0.0–0.2)	0.2 (0.1–0.3)	0.1 (0.0–0.2)	0.8 (0.5–1.1)	2.9 (2.4–3.4)	2.1 (1.6–2.5)
40–49	13.0 (12.0–14.0)	30.6 (29.3–32.0)	17.6 (16.6–18.7)	7.9 (7.0–8.8)	3.6 (3.1–4.1)	15.4 (14.4–16.4)	12.1 (11.7–12.5)	0.8 (0.6–1.1)	0.8 (0.5–1.1)	-0.2 (− 0.3--0.1)	3.0 (2.5–3.5)	8.9 (8.1–9.7)	5.9 (5.2–6.5)
50–59	30.3 (28.9–31.6)	50.8 (49.3–52.2)	20.5 (19.3–21.7)	23.2 (21.9–24.4)	3.0 (2.6–3.5)	14.6 (13.6–15.6)	11.2 (10.8–11.6)	3.1 (2.6–3.6)	2.7 (2.2–3.2)	− 0.7 (− 0.8--0.6)	5.1 (4.4–5.7)	14.5 (13.6–15.5)	9.5 (8.7–10.3)
60–69	51.1 (49.4–52.7)	66.6 (65.0–68.1)	15.5 (14.4–16.6)	30.0 (28.7–31.3)	1.3 (1.0–1.7)	6.7 (6.0–7.5)	5.4 (5.1–5.8)	7.2 (6.3–8.1)	7.5 (6.7–8.3)	0.5 (0.3–0.7)	3.2 (2.6–3.7)	13.0 (11.9–14.1)	9.9 (8.9–10.8)
70–79	65.8 (63.9–67.6)	77.1 (75.5–78.7)	11.4 (10.1–12.6)	30.0 (28.7–31.3)	0.7 (0.4–0.9)	2.4 (1.8–2.9)	2.2 (2.0–2.5)	8.6 (7.6–9.6)	11.0 (9.9–12.2)	1.8 (1.5–2.0)	2.3 (1.7–2.8)	9.7 (8.5–10.8)	7.4 (6.4–8.4)
80+	73.2 (69.8–76.6)	83.0 (80.2–85.8)	9.8 (7.7–12.0)	8.1 (7.2–8.9)	0.4 (0.0–0.8)	1.0 (0.3–1.7)	0.9 (0.8–1.1)	11.8 (9.3–14.3)	14.6 (11.7–17.4)	3.0 (2.8–3.1)	2.6 (1.4–3.8)	9.7 (7.4–11.9)	7.0 (5.0–9.0)

Values are presented as weighted % (95% confidence interval)

*Abbreviations*: *ACC/AHA* American College of Cardiology/American Heart Association, *HTN* Hypertension, *KNHANES* The Korean National Health and Nutrition Examination Survey, *KSH* Korean Society of Hypertension

When examining individual subtype of hypertension, there were diverging magnitude and direction of changes in prevalence calculated based on the two guidelines. The most marked change stemmed from increased prevalence of IDH, from 5.2% defined by the 2018 KSH guideline to 17.9% defined by the 2017 ACC/AHA guideline. Specifically, the dramatic increase was observed in young age groups 30–49 years (14.9% in age 30–39 years; 15.2% in age 40–49 years). When further stratifying by sex, young males of age 20–29 years also contributed to substantial increase, whereas older females in their fifth decade underwent considerable increase in IDH prevalence. In contrast, the changes in ISH prevalence were notably miniscule. The overall difference between the 2018 KSH and 2017 ACC/AHA guideline was 0.1%, which was relatively evenly distributed across all age groups.. However, interestingly, in middle-aged population, the prevalence of ISH actually decreased when defined by the 2017 ACC/AHA guideline; in male, such reductions were observed in age groups 40–49 years by − 0.1%, 50–59 years by − 1.4%, and 60–69 years by − 0.3% in female, in age groups 40–49 years by − 0.2% and 50–59 years by − 0.7%. Lastly, the prevalence of SDH increased from 3.5% (95% CI 3.3–3.7) defined by the 2018 KSH guideline to 11.1% defined by the 2017 ACC/AHA guideline, yielding 7.6% increase in total study population. The difference acclimated to its zenith 11.4% in 50–59 years male and 9.9% in 60–69 years female. The largest sex-discrepancy in SDH prevalence shift was observed in the youngest group, where the 2017 ACC/AHA guideline extended additional 6.3% male whereas only 0.8% female.

### Additional hypertension prevalence according to the 2017 ACC/AHA guideline

Table 3, Additional file 1: Table S1 and Additional file 1: Table S2 describe a subset of 11,336 individuals who are additionally categorized into hypertension group according to the 2017 ACC/AHA guideline from the prehypertension group by the 2018 KSH guideline—referred to as the difference of the two guidelines. Of those, 8067 individuals (76.2%) are qualified as hypertension status as IDH, 1289 individuals (8.2%) as ISH, and 1980 individuals (15.6%) as SDH. Compared to those newly classified as hypertension group as ISH or SDH subtype, the IDH counterpart encompassed significantly higher proportion of young age groups and male sex (*p* <0.0001). Moreover, it had higher proportion of current smoker and drinker (*p* <0.0001). The SDH group had the highest body mass index and the most adverse lipid profile, whereas the ISH group had the highest proportion of DM, CKD, and CVD history. Across the three subtypes, the ISH group had the highest mean 10-yr predicted ASCVD risk score (40.1% by PCE; 24.3% by KRPM), followed by the SDH group (10.6% by PCE; 9.5% by KRPM), then the IDH group (2.1% by PCE; 2.7% by KRPM).

**Table 3:** Characteristics of study participants by stage 1 hypertension subtype according to the 2017 ACC/AHA guideline based on 2007–2017 KNHANES (*n* = 11,336)

	Stage 1 hypertension by the 2017 ACC/AHA Guideline			
	Stage 1 IDH; SBP <130 mmHg and DBP ≥80 mmHg	Stage 1 ISH; SBP ≥130 mmHg and DBP <80 mmHg	Stage 1 SDH; SBP ≥130 mmHg and DBP ≥80 mmHg	*p*-value
	(*n* = 8067)	(*n* = 1289)	(*n* = 1980)	
% Study population	76.2 (75.2–77.2)	8.2 (7.6–8.8)	15.6 (14.8–16.4)	
Male sex	67.5 (66.3–68.6)	44.1 (40.7–47.4)	58.6 (56.0–61.2)	<0.0001
Age group, yrs				<0.0001
20–29	17.4 (16.3–18.6)	6.9 (4.6–9.2)	11.1 (9.0–13.2)	
30–39	25.3 (24.0–26.5)	3.6 (2.2–5.1)	12.6 (10.6–14.5)	
40–49	29.1 (27.9–30.3)	7.1 (5.0–9.1)	20.6 (18.3–22.8)	
50–59	20.9 (19.8–21.9)	19.4 (16.5–22.3)	31.1 (28.6–33.5)	
60–69	5.9 (5.4–6.3)	27.3 (24.5–30.0)	17.2 (15.4–19.0)	
70–79	1.3 (1.1–1.6)	28.4 (25.5–31.2)	6.6 (5.5–7.6)	
80+	0.2 (0.1–0.2)	7.3 (5.7–8.9)	0.9 (0.5–1.3)	
Smoking status				<0.0001
Non-smoker	42.9 (41.6–44.2)	59.0 (55.5–62.5)	50.7 (48.0–53.4)	
Previous smoker	17.9 (16.8–18.9)	17.4 (14.9–20.0)	18.4 (16.2–20.5)	
Current smoker	39.3 (37.9–40.6)	23.6 (20.5–26.6)	30.9 (28.5–33.4)	
Alcohol intake				<0.0001
Non-drinker	6.2 (5.6–6.8)	21.3 (18.7–23.8)	11.4 (9.8–12.9)	
Former drinker	25.2 (24.0–26.3)	35.1 (31.8–38.4)	27.1 (24.8–29.4)	
Current drinker	68.6 (67.4–69.8)	43.6 (40.2–47.0)	61.6 (58.9–64.2)	
Regular exercise				<0.0001
Yes	30.4 (29.0–31.8)	25.8 (22.3–29.3)	30.4 (27.8–33.0)	
No	69.6 (68.2–71.0)	74.2 (70.7–77.7)	69.6 (67.0–72.2)	
BMI, kg/m^2^	24.4 (24.3–24.5)	23.8 (23.5–24.0)	24.8 (24.6–25.0)	<0.0001
Total cholesterol, mg/dL	195.7 (194.7–196.6)	192.4 (190.0–194.8)	199.6 (197.7–201.6)	<0.0001
HDL cholesterol, mg/dL	49.2 (48.9–49.5)	46.8 (48.0–49.5)	49.2 (48.5–49.9)	<0.0001
Triglyceride, mg/dL	158.9 (154.6–163.2)	136.2 (129.1–143.3)	165.3 (158.3–172.4)	<0.0001
Lipid-lowering agent intake	2.2 (1.8–2.5)	7.5 (5.9–9.2)	3.7 (2.8–4.5)	<0.0001
Fasting glucose, mg/dL	98.0 (97.4–98.6)	104.7 (102.9–106.5)	101.3 (100.1–102.5)	<0.0001
GFR, ml/min/1.73 m^2^	97.6 (97.2–98.1)	87.9 (86.7–89.0)	94.2 (93.4–95.0)	<0.0001
Diabetes mellitus	6.4 (5.7–7.0)	18.2 (15.5–20.8)	9.2 (7.7–10.7)	<0.0001
Chronic Kidney Disease	0.5 (0.4–0.6)	5.1 (3.7–6.4)	1.3 (0.8–1.8)	<0.0001
Mean 10-yr predicted CVD risk^*^				
High risk,^*^%, PCE	2.1 (1.8–2.4)	40.1 (36.7–43.5)	10.6 (9.3–12.0)	<0.0001
High risk, ^*^%, KRPM	2.7 (2.3–3.0)	24.3 (21.6–27.0)	9.5 (8.1–10.9)	<0.0001
History of CVD	3.1 (2.6–3.6)	11.2 (9.0–13.4)	5.4 (4.1–6.7)	<0.0001

Values are presented as % or mean (95% confidence interval)

*P*-values are derived from analysis of variance (ANOVA) for multiple comparison across the three subtype groups

*Abbreviations*: *ACC/AHA* American College of Cardiology/American Heart Association, *BMI* Body mass index, *CI* Confidence interval, *CKD* Chronic kidney disease, *CVD* Cardiovascular disease, *GFR* Glomerular filtration rate, *HDL* High-density lipoprotein, *IDH* Isolated diastolic hypertension, *ISH* Isolated systolic hypertension, *KRPM* Korean Risk Prediction Model, *KSH* Korean Society of Hypertension, *PCE* Pooled Cohort Equation, *SDH* Systolic diastolic hypertension

^*^10-yr risk was calculated among adults without a history of CVD

^*^High risk defined as a 10-year predicted cardiovascular disease risk ≥10% or history of CVD

## Discussion

In this study of nationally representative samples, we extended the conventional assay of hypertension prevalence by assessing its difference using the two contemporary diagnostic criteria. An important advance of this study was how we further examined the subtype composition that attributed to overall differences, separately by sex and age groups. Overall, the prevalence of all untreated hypertension was markedly higher by the 2017 ACC/AHA guideline due to the lowered BP cutoff, which was primarily manifested through increase in IDH prevalence, particularly in younger population. This predominant make-up of IDH is further highlighted by the considerable stagnancy in ISH prevalence despite the lowered SBP cutoff and moderate increase in SDH prevalence, presumably resulting from the lowered diastolic cutoff.

Our results are in accordance with the established BP pathophysiology over lifespan. The natural trajectory of BP increases until the fifth or sixth decade of life, subsequently followed by a gradual decline of DBP whilst a continued elevation of SBP; such phenomena are immune to demographics, treatment status, and physician practices [29]. Consequently, such diverging course of SBP and DBP supports observations [30, 31] noted in Korean population, in which IDH was the most frequently found among young participants, whereas ISH was more commonplace in the older counterpart. Such was also observed in Chinese population, where the age-adjusted hypertension prevalence of total study population was 20.9%, which was comprised of IDH, 4.44%; ISH, 3.30%; SDH, 4.11%; and the remainder, treated [32]. Moreover, the age-differential proportion of subtypes among the treated individuals may also explain the high proportion of IDH observed in our study. According to the Korea Hypertension Fact Sheet 2018 [7], young adults had substantially lower hypertension awareness, treatment, and control rate compared to their older counterparts. Then, assuming a large proportion of SDH is likely to be embodied by elderly population with higher likelihood of treatment, hypertension proportion may be heterogeneous between untreated and treated individuals, thereby explaining the pronounced IDH prevalence in our data of untreated individuals.

Our findings also concur with the pattern of increased hypertension prevalence in adherence to the 2017 ACC/AHA guideline, reported in Muntner et al.’s assay of potential US population impact from the 2017 ACC/AHA guideline [33]. Consistent with our findings, the overall hypertension prevalence was 31.9% (versus 25.9% in our study) by the JNC 7 guidelines, with 13.7% elevation to 45.6% (versus 46.3%, likewise) by the 2017 ACC/AHA guideline among untreated participants; such increase was present for all age, sex, race/ethnicity, and CVD risk subgroups except those with a very low 10-year CVD risk (<5%).

The differences in hypertension subtype prevalence by the two guidelines were differentially manifested by age distributions. Specifically, the majority of the added prevalence originated from age 20–49 years qualifying as IDH then as SDH with the lowered BP cutoff by the 2017 ACC/AHA guideline. This drastic growth in IDH prevalence is of particular concern. Fundamentally, the 2017 ACC/AHA guideline’s shift to lowered BP cutoff was based on evidence from epidemiologic studies [10, 11] and randomized trials [34–37]; they demonstrated that even mildly elevated BP (defined as prehypertension by the 2018 KSH guideline) would increase risk for adverse heart-related outcomes, and lowering of SBP below 130 mmHg would reduce CVD risk. A previous meta-analysis showed that lowering of usual SBP by 20-mmHg or DBP by 10-mmHg was associated with halved risk for all stroke and cerebral heart disease mortality, with indefinite lower threshold [9]. However, the clinical significance and utility of DBP for CVD risk prediction have been recently pondered upon [16]. Previous findings merit SBP as more robust and consistent BP component for later cardiovascular risk, whereas DBP showed inconsistent association after adjustment for or stratification by SBP [16]. Moreover, there are perplexities regarding the validity of incorporating DBP threshold in defining hypertension and in referring for initiation of pharmaceutical treatment [13, 38, 39], as most of the randomized trials’ eligibility criteria have historically been centered around SBP [35, 36, 40].

Yet, despite diversified perspectives surrounding the benefits of detecting and treating mildly elevated DBP in young people (particularly in those without target organ comorbidity or near-zero 10-year ASCVD risk), we remain reserved to ascertain its benign nature. Majority of the aforementioned studies that examined the prospective association between BP and future CVD incidence and risk have primarily, if not solely, included middle-aged to elderly subjects, whom the likelihood of high DBP is inevitably lower than that of SBP. Furthermore, even if they had included younger population, the higher 10-year ASCVD risk levels embodied by older subjects would dilute the relative risks presented in the younger population. Evidence also shows that high DBP by the 2017 ACC/AHA guideline did have increased risk for future CVD, after adjusting for age, sex, body mass index, socioeconomic status, smoking history, statin use and various competing comorbidity (hazards ratio = 1.12, 95% CI 1.05–1.19) [16]. Considering the trajectory and associated risk of early onset hypertension are maintained until the later life, further studies are warranted to narrow scope on young populations’ BP and ASCVD risk level distributions for accurate assessment of their absolute, relative, and population attributable risks [41, 42]. Lastly, the recently released European Society of Cardiology/European Society of Hypertension (ESC/ESH) guideline also appears in favor of stringent BP control compared to its previous version by recommending drug treatment for high normal BP (130–139/85–89 mmHg) with high underlying CVD risk [43]. In the absence of long-term randomized trials on these subpopulations and with established findings on prolonged aftermath of hypertension diagnosed during young adulthood, our estimates pave epidemiological ground to explore potential risk of IDH in young age.

Our study has several notable strengths. To the extent of our knowledge, this is the first study to examine all hypertension and its subtype prevalence by the two contemporary guidelines embodying different diagnostic criteria and to assess their differences in sex- and age-specific manner. With KNHANES’ collection of wide range and rich depth of information on nationally representative samples, our findings can adequately capture contemporary population health status across various stages and types of hypertension. In addition, the examiners have used standardized methods and validated tools to minimize potential measurement bias and errors.

Despite robust data source and meticulous examination, limitations exist in the inherent characteristics of the study population and design. Since the KNHANES is essentially composed of homogeneous Korean ethnicity, extrapolation of our findings to different race should be sought with consideration for distinctive population structure, healthcare system, and individual biology and health behaviors. Moreover, since we have excluded participants who are currently taking antihypertensive medication, our results cannot be generalized irrespective of treatment status, as the treatment initiation and adherence rate are known to significantly differ by demographics [44]. In terms of data collection, although we have adopted a mean from consecutive measurements, the nature of single-occasion BP measurements are subjected to variability. Another potential source of misclassification bias may arise from white coat hypertension, a condition in which patients experience persistent high BP levels when they are measured in clinical surrounding or at a presence of physician [45]. Future studies using repeated and sporadic measurements from both clinic and ambulatory settings are encouraged for more stable representation of BP levels. Lastly, because the information regarding demographics and health-related behaviors, including the formal diagnosis of hypertension and frequency of antihypertensive intake, were obtained via self-report, recall bias cannot be ruled out.

## Conclusions

In conclusion, changes in each hypertension subtype prevalence make differential contribution to the additionally classified cases according to the 2017 ACC/AHA guideline compared to the 2018 KSH guideline, and the degree of such contributions is distinguishable by sex and age. Whereas the changes in ISH prevalence is, overall, negligible and moderate increases in SDH prevalence are observed in middle-aged population, the marked increase in IDH prevalence among young age groups highlights the overall increase in all hypertension prevalence. Despite seemingly-ameliorative 10-year ASCVD risk levels estimated in young individuals, we currently lack prospective data with sufficient time horizons to infer clinical implications of lowered systolic and diastolic cutoffs. Therefore, irrespective of the baseline risk, future epidemiological studies should surveil subclinical ASCVD incidence and clinical trials should investigate the benefits of long-term primary prevention, specifically targeting the young population. Until then, persistent and comprehensive BP management should be sought, with discretion, but with tenacity, regardless of degree of BP elevation in any age group.

## Supplementary information

**Additional file 1: Table S1.** Number of study participants meeting the definition of hypertension and its subtypes. **Table S2.** Proportion of stage 1 hypertension subtype by the 2017 ACC/AHA guideline.

## Data Availability

Survey data are publicly available at (https://knhanes.cdc.go.kr/main.do). All data from KNHANES IV-VII are coded and freely available.

## Abbreviations

ACC: American College of Cardiology
AHA: American Heart Association
ASCVD: Atherosclerotic cardiovascular diseases
BP: Blood pressure
CI: Confidence interval
CKD: Chronic kidney disease
CVD: Cardiovascular disease
DBP: Diastolic blood pressure
DM: Diabetes mellitus
IDH: Isolated diastolic hypertension
ISH: Isolated systolic hypertension
JNC 7: Joint National Committee on Prevention, Detection, Evaluation and Treatment of High Blood Pressure
KNHANES: Korea National Health and Nutrition Examination Survey
KRPM: Korean Risk Prediction Model
KSH: Korean Society of Hypertension
PCE: Pooled Cohort Equation
SBP: Systolic blood pressure
SDH: Systolic diastolic hypertension
