# Supplementary material for: Differences in prevalence of hypertension subtypes according to the 2018 Korean Society of Hypertension and 2017 American College of Cardiology/American Heart Association guidelines: The Korean National Health and Nutrition Examination Survey, 2007–2017 (KNHANES IV-VII)
Source: Clin Hypertens. 2019 Dec 1;25:26. doi: 10.1186/s40885-019-0129-5 (PMC6885311; doi:10.1186/s40885-019-0129-5)
Supplement: Supplementary file 1 — Additional file 1: Table S1. Number of study participants meeting the definition of hypertension and its subtypes. Table S2. Proportion of stage 1 hypertension subtype by the 2017 ACC/AHA guideline. [file 40885_2019_129_MOESM1_ESM.docx]

| Table S1. Number of study participants meeting the definition of hypertension and its subtypes | | | | | | | | | | | | | | | | | | | | | |
| --- | --- | --- | --- | --- | --- | --- | --- | --- | --- | --- | --- | --- | --- | --- | --- | --- | --- | --- | --- | --- | --- |
|  | Total | 2017 ACC/AHA Guideline | | | | | |  | 2018 KSH Guideline | | | | | |  | Difference (2017 ACC/AHA, But Not 2018 KSH) | | | | | |
|  |  | All HTN | Untreated HTN | | | | Treated |  | All HTN | Untreated HTN | | | | Treated |  | All HTN | Untreated HTN | | | | Treated |
|  |  |  | All | IDH | ISH | SDH | HTN |  |  | All | IDH | ISH | SDH | HTN |  |  | All | IDH | ISH | SDH | HTN |
| Total | 59767 | 29762 | 18065 | 9191 | 2082 | 6743 | 11697 |  | 18463 | 6766 | 2592 | 2007 | 2081 | 11697 |  | 11299 | 11299 | 6599 | 75 | 4662 | 0 |
| Age group, yrs |  |  |  |  |  |  |  |  |  |  |  |  |  |  |  |  |  |  |  |  |  |
| 20-29 | 6765 | 1382 | 1371 | 1071 | 40 | 260 | 11 |  | 284 | 273 | 209 | 19 | 45 | 11 |  | 1098 | 1098 | 862 | 21 | 215 | 0 |
| 30-39 | 10985 | 3155 | 3021 | 2251 | 34 | 735 | 134 |  | 1010 | 876 | 643 | 24 | 208 | 134 |  | 2145 | 2145 | 1608 | 10 | 527 | 0 |
| 40-49 | 11444 | 4895 | 4114 | 2660 | 63 | 1389 | 781 |  | 2249 | 1468 | 883 | 82 | 496 | 781 |  | 2646 | 2646 | 1777 | -19 | 893 | 0 |
| 50-59 | 11465 | 6664 | 4337 | 2073 | 265 | 1991 | 2327 |  | 4002 | 1675 | 599 | 365 | 693 | 2327 |  | 2662 | 2662 | 1474 | -100 | 1298 | 0 |
| 60-69 | 10272 | 7012 | 3016 | 867 | 688 | 1444 | 3996 |  | 5303 | 1307 | 192 | 684 | 405 | 3996 |  | 1709 | 1709 | 675 | 4 | 1039 | 0 |
| 70-79 | 7325 | 5462 | 1819 | 242 | 772 | 792 | 3643 |  | 4581 | 938 | 59 | 656 | 201 | 3643 |  | 881 | 881 | 183 | 116 | 591 | 0 |
| 80+ | 1511 | 1192 | 387 | 27 | 220 | 132 | 805 |  | 1034 | 229 | 7 | 177 | 33 | 805 |  | 158 | 158 | 20 | 43 | 99 | 0 |
|  |  |  |  |  |  |  |  |  |  |  |  |  |  |  |  |  |  |  |  |  |  |
| Male | 25750 | 15305 | 10264 | 5662 | 849 | 3735 | 5041 |  | 9005 | 3964 | 1892 | 843 | 1202 | 5041 |  | 6300 | 6300 | 3770 | 6 | 2533 | 0 |
| Age group, yrs |  |  |  |  |  |  |  |  |  |  |  |  |  |  |  |  |  |  |  |  |  |
| 20-29 | 2950 | 1022 | 1013 | 753 | 37 | 223 | 9 |  | 233 | 224 | 168 | 18 | 38 | 9 |  | 789 | 789 | 585 | 19 | 185 | 0 |
| 30-39 | 4613 | 2190 | 2097 | 1525 | 20 | 551 | 93 |  | 795 | 702 | 526 | 17 | 158 | 93 |  | 1395 | 1395 | 999 | 3 | 393 | 0 |
| 40-49 | 4969 | 2918 | 2488 | 1642 | 18 | 828 | 430 |  | 1426 | 996 | 661 | 27 | 304 | 430 |  | 1492 | 1492 | 981 | -9 | 524 | 0 |
| 50-59 | 4871 | 3290 | 2214 | 1116 | 88 | 1009 | 1076 |  | 1982 | 906 | 390 | 151 | 363 | 1076 |  | 1308 | 1308 | 726 | -63 | 646 | 0 |
| 60-69 | 4586 | 3203 | 1461 | 482 | 266 | 706 | 1742 |  | 2367 | 625 | 115 | 278 | 224 | 1742 |  | 836 | 836 | 367 | -12 | 482 | 0 |
| 70-79 | 3191 | 2268 | 843 | 129 | 333 | 376 | 1425 |  | 1848 | 423 | 28 | 281 | 106 | 1425 |  | 420 | 420 | 101 | 52 | 270 | 0 |
| 80+ | 570 | 414 | 148 | 15 | 87 | 42 | 266 |  | 354 | 88 | 4 | 71 | 9 | 266 |  | 60 | 60 | 11 | 16 | 33 | 0 |
|  |  |  |  |  |  |  |  |  |  |  |  |  |  |  |  |  |  |  |  |  |  |
| Female | 34017 | 14457 | 7801 | 3529 | 1233 | 3008 | 6656 |  | 9458 | 2802 | 700 | 1164 | 879 | 6656 |  | 4999 | 4999 | 2829 | 69 | 2129 | 0 |
| Age group, yrs |  |  |  |  |  |  |  |  |  |  |  |  |  |  |  |  |  |  |  |  |  |
| 20-29 | 3815 | 360 | 358 | 318 | 3 | 37 | 2 |  | 51 | 49 | 41 | 1 | 7 | 2 |  | 309 | 309 | 277 | 2 | 30 | 0 |
| 30-39 | 6372 | 965 | 924 | 726 | 14 | 184 | 41 |  | 215 | 174 | 117 | 7 | 50 | 41 |  | 750 | 750 | 609 | 7 | 134 | 0 |
| 40-49 | 6475 | 1977 | 1626 | 1018 | 45 | 561 | 351 |  | 826 | 472 | 222 | 55 | 192 | 351 |  | 1151 | 1151 | 796 | -10 | 369 | 0 |
| 50-59 | 6594 | 3374 | 2123 | 957 | 177 | 982 | 1251 |  | 2020 | 769 | 209 | 214 | 330 | 1251 |  | 1354 | 1354 | 748 | -37 | 652 | 0 |
| 60-69 | 5686 | 3809 | 1555 | 385 | 422 | 738 | 2254 |  | 2936 | 682 | 77 | 406 | 181 | 2254 |  | 873 | 873 | 308 | 16 | 557 | 0 |
| 70-79 | 4134 | 3194 | 976 | 113 | 439 | 416 | 2218 |  | 2733 | 515 | 31 | 375 | 95 | 2218 |  | 461 | 461 | 82 | 64 | 321 | 0 |
| 80+ | 941 | 778 | 239 | 12 | 133 | 90 | 539 |  | 680 | 141 | 3 | 106 | 24 | 539 |  | 98 | 98 | 9 | 27 | 66 | 0 |
| Values are presented as number. | | | | | | | | | | | | | | | | | | | | | |
| Abbreviations: ACC/AHA, American College of Cardiology/American Heart Association; HTN, hypertension; KNHANES, the Korean National Health and Nutrition Examination Survey; | | | | | | | | | | | | | | | | | | | | | |
| KSH, Korean Society of Hypertension; IDH, isolated diastolic hypertension; ISH, isolated systolic hypertension; SDH, systolic diastolic hypertension | | | | | | | | | | | | | | | | | | | | | |

| Table S2. Proportion of stage 1 hypertension subtype by the 2017 ACC/AHA guideline | | | | | |
| --- | --- | --- | --- | --- | --- |
|  | 2018 KSH Guideline |  | Stage 1 hypertension by the 2017 ACC/AHA Guideline | | |
|  | Prehypertension; |  | Stage 1 IDH; | Stage 1 ISH; | Stage 1 SDH; |
|  | SBP 130-139 mmHg |  | SBP <130 mmHg | SBP ≥130 mmHg | SBP ≥130 mmHg |
|  | or |  | and | and | and |
|  | DBP 80-89 mmHg |  | DBP ≥80 mmHg | DBP <80 mmHg | DBP ≥80 mmHg |
|  | (n=11,336) |  | (n=8,067) | (n=1,289) | (n=1,980) |
| Male | 6,309 |  | 4796 (76.0) | 525 (8.3) | 988 (15.7) |
| Age group, yrs |  |  |  |  |  |
| 20-29 | 789 |  | 653 (82.8) | 35 (4.4) | 101 (12.8) |
| 30-39 | 1,395 |  | 1255 (90.0) | 17 (1.2) | 123 (8.8) |
| 40-49 | 1,496 |  | 1331 (89.0) | 14 (0.9) | 151 (10.1) |
| 50-59 | 1,309 |  | 967 (73.9) | 62 (4.7) | 280 (21.4) |
| 60-69 | 837 |  | 454 (54.2) | 161 (19.2) | 222 (26.5) |
| 70-79 | 423 |  | 123 (29.1) | 196 (46.3) | 104 (24.6) |
| 80+ | 60 |  | 13 (21.7) | 40 (66.7) | 7 (11.7) |
|  |  |  |  |  |  |
| Female | 5,027 |  | 3271 (65.1) | 764 (15.2) | 992 (19.7) |
| Age group, yrs |  |  |  |  |  |
| 20-29 | 309 |  | 291 (94.2) | 3 (1.0) | 15 (4.9) |
| 30-39 | 750 |  | 672 (89.6) | 13 (1.7) | 65 (8.7) |
| 40-49 | 1,155 |  | 936 (81.0) | 40 (3.5) | 179 (15.5) |
| 50-59 | 1,363 |  | 894 (65.6) | 139 (10.2) | 330 (24.2) |
| 60-69 | 881 |  | 364 (41.3) | 259 (29.4) | 258 (29.3) |
| 70-79 | 467 |  | 103 (22.1) | 244 (52.2) | 120 (25.7) |
| 80+ | 102 |  | 11 (10.8) | 66 (64.7) | 25 (24.5) |
| Values are presented as number (% row total). | | | | | |
| Abbreviations: ACC/AHA, American College of Cardiology/American Heart Association; IDH, isolated diastolic hypertension; | | | | | |
| ISH, isolated systolic hypertension, KSH, Korean Society of Hypertension; SDH, systolic diastolic hypertension | | | | | |
